# Rapid alignment of nanotomography data using joint iterative reconstruction and reprojection

**DOI:** 10.1038/s41598-017-12141-9

**Published:** 2017-09-18

**Authors:** Doğa Gürsoy, Young P. Hong, Kuan He, Karl Hujsak, Seunghwan Yoo, Si Chen, Yue Li, Mingyuan Ge, Lisa M. Miller, Yong S. Chu, Vincent De Andrade, Kai He, Oliver Cossairt, Aggelos K. Katsaggelos, Chris Jacobsen

**Affiliations:** 1 Advanced Photon Source, Argonne National Laboratory, 9700 South Cass Avenue, Lemont, IL 60439 USA; 2 Department of Electrical Engineering and Computer Science, Northwestern University, 2145 Sheridan Road, Evanston, IL 60208 USA; 3 Department of Physics and Astronomy, Northwestern University, 2145 Sheridan Road, Evanston, IL 60208 USA; 4 Department of Materials Science and Engineering, Northwestern University, 2220 Campus Drive, Evanston, IL 60208 USA; 5 National Synchrotron Light Source-II, Brookhaven National Laboratory, Upton, NY 11967 USA; 6 Chemistry of Life Processes Institute, Northwestern University, 2170 Campus Drive, Evanston, IL 60208 USA

## Abstract

As x-ray and electron tomography is pushed further into the nanoscale, the limitations of rotation stages become more apparent, leading to challenges in the alignment of the acquired projection images. Here we present an approach for rapid post-acquisition alignment of these projections to obtain high quality three-dimensional images. Our approach is based on a joint estimation of alignment errors, and the object, using an iterative refinement procedure. With simulated data where we know the alignment error of each projection image, our approach shows a residual alignment error that is a factor of a thousand smaller, and it reaches the same error level in the reconstructed image in less than half the number of iterations. We then show its application to experimental data in x-ray and electron nanotomography.

## Introduction

**Figure 1:**
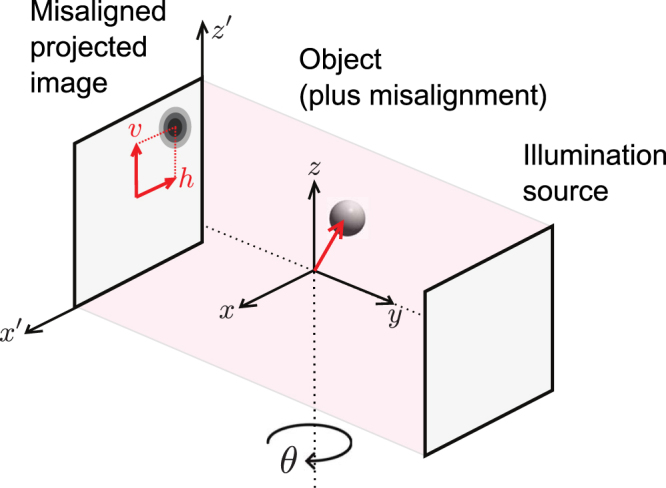
Schematic of the tomographic data acquisition process. Misalignment of the object in 3D space leads to a different 2D translation error (*h*, *v*) on each projection image taken at tilt angle *θ*.

In tomography, a series of two-dimensional (2D) projections are acquired as a three-dimensional (3D) object is rotated about one or more axes, after which a 3D reconstruction of the object is obtained. Implicit in the approach is the idea that the only differences between the projections are the known rotational angles, with no additional translations or other motions of the object (Fig. 1). This condition is easy to meet in traditional forms of tomography at millimeter length scales when using precision rotation stages; however, it becomes challenging at sub-100 nm length scale of x-ray or electron nanotomography, where one uses high resolution microscopes to obtain 2D projections revealing nanoscale morphology. At these fine length scales, imperfections in rotation stage motion (often called runout errors, or spindle errors) become more noticeable. While the synchronous (reproducible) component of runout errors can be characterized in advance^1^ and corrected for, this is not the case for asynchronous errors due to imperfect roundness of bearings and other factors. These random runout errors are typically at the level of about 15 nm in today’s best rotation stages. A limited number of microscopes incorporate sophisticated metrology systems to measure and correct for runout error within a certain limit^2–5^; however, experimental constraints such as limited working distance, or cryogenic specimen conditions, make systems of this type the exception rather than the rule.

There is a class of “iterative reprojection” techniques which have been demonstrated to provide a good solution to the uncalibrated tomography problem through a succession of iterative alignment steps following tomographic reconstruction steps. To date, most prior work in this area has relied on filtered backprojection as the tomographic reconstruction algorithm because of its speed and efficiency. However, because many x-ray microscopes take more time to acquire projection images of large objects than electron microscopes do of smaller objects, it is not uncommon in x-ray microscopy to be limited to tomographic datasets with a smaller number of projections than one would have demanded by strict adherence to the Crowther criterion^6^. In this case of limited number and range of projection angles, iterative reconstruction algorithms such as algebraic^7,8^ or maximum likelihood^9^ methods are preferred, because they allow for including a priori information about the data and object, which is essential in obtaining satisfactory reconstructions. Therefore, several authors have developed approaches to using similar iterative reconstruction methods at each step of the reprojection iteration^10–13^. However, iterations within iterations are inefficient, and the compute-intensive nature of these iterative methods makes their use less practical for large x-ray datasets.

We report here a new variant to iterative reprojection tomography alignment with a key advance: rather than run *N* cycles of iterative tomographic reconstruction for each of the *M* steps of iterative reprojection sequentially (Algorithm 1), we carry out one joint reprojection step after each iteration of the reconstruction algorithm (Algorithm 2). Because the computational complexity of the proposed method (Algorithm 2) is ${\mathscr{O}}(N)$ rather than ${\mathscr{O}}(NM)$ with *N* and *M* being the number of reconstruction iterate and alignment iterate, this has the potential for significantly speeding up the combined alignment and reconstruction process. It also avoids the possibility of having separate algorithmic steps traveling for long distances in one direction in parameter space while another step may need to move in an orthogonal direction. We demonstrate the use of this approach in x-ray and electron microscopy using both simulated and experimental datasets, where we have obtained improved reconstructions from difficult-to-align tomographic datasets. We show by example that the proposed joint reprojection algorithm (Algorithm 2) can produce such improved reconstructions with higher convergence rates than the conventional sequential algorithm (Algorithm 1), including with noisy and undersampled datasets. This is shown both in selected 2D slices, and in a 3D rendering.

## Related Work

A wide range of approaches for projection alignment are employed in electron tomography^14^. The most common approach is to use cross-correlation between projections acquired at adjacent rotation angles^15–18^, or correlation of vertical variations in the mass of the sample^19^. However, two features in 3D space can have their apparent separation change in projections as a function of rotation angle, leading to ambiguities on which feature dominates the cross-correlation. These ambiguities exponentiate as the number of features are increased, or as the rotation angles between projection images are widened. As a result, while cross-correlation alignment can remove frame-to-frame “jitter” in tomographic datasets, it cannot be relied upon to find a common rotation axis for complete set of projections^20^.

When specimens are mounted within semi-transparent capillary holders, one can use high-contrast capillary edges to correct for jitter^21^. An alternative approach is to place fiducial markers such as small gold beads^22^ or silica spheres^23^ on the specimen mount or directly on the specimen, and identify them either manually or automatically^24,25^; their positions can then be used to correct for alignment errors^26^. This approach is quite successful, and is frequently employed; however, it comes at the cost of adding objects that can complicate sample preparation, obscure specimen features in certain projections, and add material that may complicate analytical methods such as the analysis of fluorescent x-rays.

For those situations where the addition of fiducial marker materials is problematic, one can instead use a variety of feature detection schemes to identify marker positions intrinsic to the specimen, after which various alignment procedures are applied^27–30^ including the use of bundle adjustment^31^. Among these natural feature selection schemes are object corner detection^32^, wavelet-based detection^33^, Canny edge detection^34^, feature curvature detection^35^, or common-line approach for registration of features in Fourier space^36^. One can use Markov random fields^37^ or the Scale-Invariant Feature Transform (SIFT)^38,39^ to refine the correspondence of features throughout the tomographic dataset. Finally, in the much simpler case of very sparse and local features, one can simply fit sinusoidal curves onto features in the sinogram representation of a set of line projections, and shift projections onto the fitted sinusoid curve^40^.

These methods are widely employed with good success in electron tomography, where the mean free path for inelastic scattering is often in the 100–200 nm range even for biological specimens with low atomic number, so that it is rare to study samples thicker than about 1 $\mu$ m (high angle dark field can allow scanning transmission electron microscopes, or STEMs, to image somewhat thicker materials^41,42^). The situation can be more challenging in nanotomography with x-ray microscopes, where the great penetration of x-rays means that samples tens of micrometers or more in size can be studied^43–48^. This freedom to work with larger specimens means that while feature-based alignment can still be employed for imaging thin specimens with low contrast^10,43^, in STEM tomography and especially in hard x-ray nanotomography it becomes increasingly challenging to track fiducials or intrinsic features due to the overlap of a large number of features in depth as seen from any one projection.

In all of the above techniques, the primary strategy is to perform an alignment that is as accurate as possible *before* tomographic reconstruction. In the last few decades, a new set of automatic alignment techniques have been introduced based on a “bootstrap” process^49^, now commonly referred to as “iterative reprojection”. These techniques attempt to achieve *simultaneous alignment and reconstruction* through an iterative refinement process. They are based on the fact that the measurement process (forward model) and object reconstruction (inverse model) should be consistent only for a correct alignment geometry. Already in its initial implementation for electron tomography^49^, a multiscale approach of the method has been used, where first a downsampled version of the projections is used to generate a lower resolution 3D object reconstruction for a first pass of alignment; this first pass with a smaller dataset can align large features in images and works quickly, after which one can improve the alignment at higher resolution until finally the full resolution dataset is used^49,50^. A variation on this approach is to generate a low-quality object reconstruction from a single projection and then use all the remaining projections for another object reconstruction, and to then align these 3D objects^51^. One can use projection cross-correlation and the common-line approach for an initial alignment so as to improve convergence times^52^. Within an optimization framework, iterative reprojection can incorporate a variety of criteria to seek optimal alignment parameters, including contrast maximization in the overall image^49^ or in sub-tomogram features^53^, cross-correlation of reprojection and original projection images^54^, and for cost function reduction a quasi-Newton distance minimization^55^ or a Levenberg-Marquardt distance minimization^56^. While initially developed for electron nanotomography, iterative reprojection schemes have also been applied in x-ray microscopy^10,57^ and with commercial x-ray microtomography systems^58,59^. As was noted above, all of these prior approaches used variations on Algorithm 1, whereas our approach using Algorithm 2 produces faster convergence rates and more robust reconstructions, and can yield better accuracy especially in the case of tomograms with a limited set of projection angles.

## Results and Discussions

### Noise studies with simulated data

**Figure 2:**
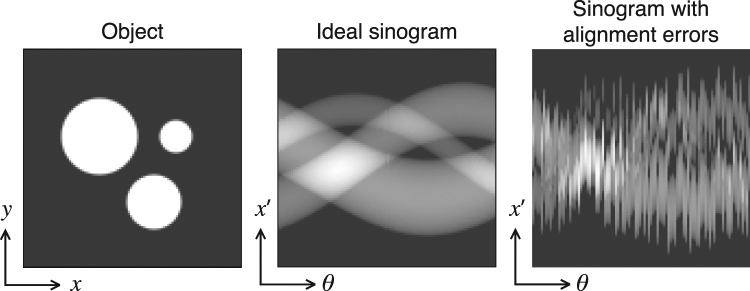
A transverse slice of the analytical 3D simulation phantom (left), ideal sinogram with no translation errors corresponding to the phantom (middle), and the sinogram after random shifts are applied (right). The sinogram is computed from 100 projection images of the phantom, with tilt angles uniformly ranging from 0 to 180 degrees for a 100 × 100 pixel area detector.

We applied the proposed joint algorithm on the simulated data shown in Fig. 2 with different noise levels. We generated an analytical phantom for evaluating the algorithm performance. The phantom consists of three spherical objects with various radii. We computed 100 projection images of the phantom with tilt angles uniformly ranging from 0 to 180 degrees for a 100 × 100 pixel area detector. We then simulated jitter in the measurement process by randomly translating the projections. The parameters for both transverse and axial shifts were generated independently from a uniform distribution $U(-10,\mathrm{10)}$ pixels in extent for each axis and for each projection. In other words, we randomly translated each projection image by a maximum of 10 pixels both in the transverse (to the axis of rotation) and axial (along the direction of the axis of rotation) directions. This corresponds to 10% jitter of the complete field of view (for a projection image of 100 × 100 size), and is typical for most nanoscale x-ray fluorescence tomography datasets. Finally, we added Gaussian noise to measurements for simulating various experimental conditions and evaluating algorithm robustness. Four different noise levels (i.e., 0%, 5%, 10% and 20%) were used, where the values in parenthesis mean that the standard deviation of noise was selected as a percentage of the maximum value of the signal in the projections.

**Figure 3:**
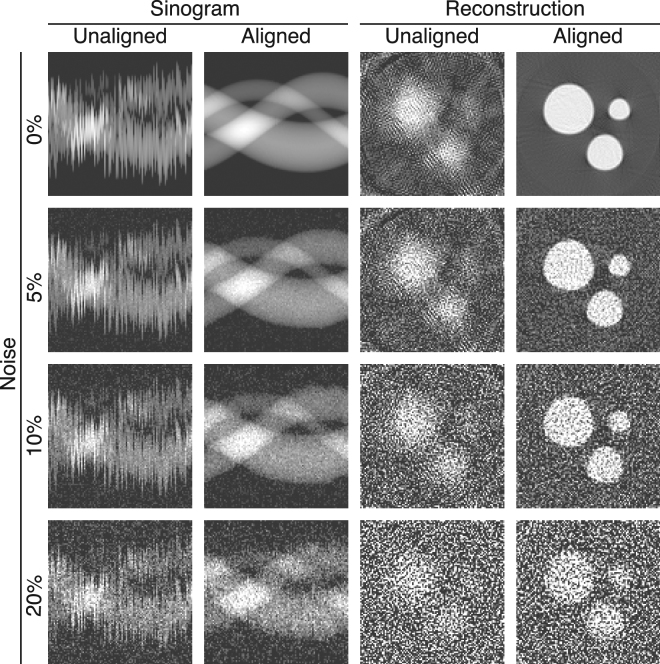
Joint reprojection/reconstruction results on the simulated 3D phantom shown in Fig. 2. Alignment errors distributed uniformly over a range of −10 to +10 pixels were applied to each projection, after which an aligned reconstruction was obtained. At left are shown the sinograms of the (input) unaligned and (output) data, and at right are shown tomographic reconstructions of a slice of the 3D object for both (input) unaligned and (output) aligned data. Our approach produces good results even with additive noise corresponding to 20% of the maximum signal level.

For joint alignment and reconstruction, we used the simultaneous iterative reconstruction technique (SIRT)^9^ for the reconstruction step at each iteration of the joint algorithm. The unaligned data and the corresponding reconstructions after convergence of the algorithm are presented in Fig. 3 together with the aligned data and reconstructions. Our joint algorithm could generate satisfactory recovery of all three spheres even for high levels of noise up to 20%, while unaligned data failed to resolve the smallest sphere, and only recovered blurred versions of the larger spheres.

**Figure 4:**
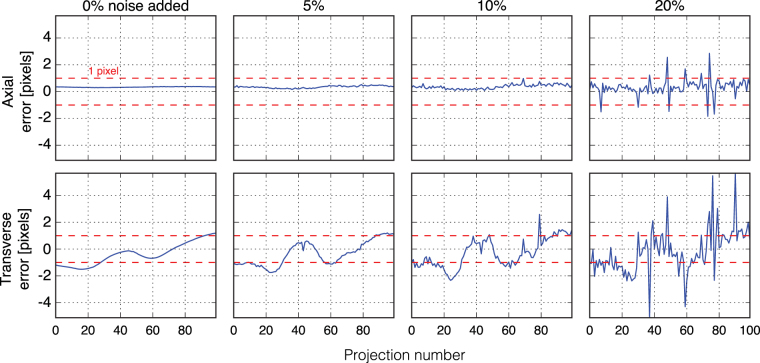
Estimation errors in image pixel units between true and recovered alignment parameters for both transverse (top row) and axial (bottom row) shifts and for different measurement noise levels. The estimation errors are calculated as the mean squared error (MSE) of the magnitude (L2-norm) of the translation vectors. The red dashed lines indicate the single pixel limit, where a shift of zero is known for our simulated dataset (Fig. 2).

The estimation errors between the true and recovered alignment parameters for both transverse and axial shifts for each projection image are plotted in Fig. 4. The dashed red lines indicate the single pixel bound. The joint algorithm could satisfactorily estimate all axial shifts for up to 10% noise (and most of them for 20% noise) at sub-pixel accuracy. The estimation accuracy is slightly worse for transverse shifts, and as expected, drops with increasing noise levels. We also observed some correlation between axial and transverse estimations. Basically, estimation of the transverse and axial shifts are usually both affected to varying degree, possibly from the image noise or other imaging artifacts appearing in a single projection image.

### Comparative studies

**Figure 5:**
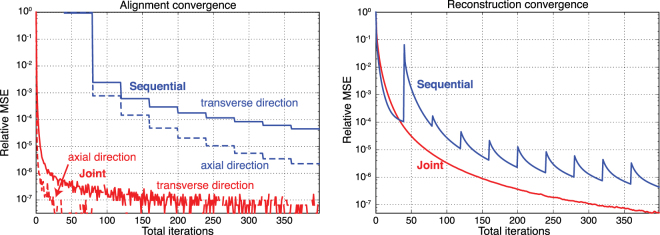
Convergence plots of the sequential (Algorithm 1) and our joint (Algorithm 2) iterative reprojection approach as applied to our simulated dataset of Fig. 2. We show at left the mean square error (MSE) for the estimation alignment shifts in pixels, while at right is shown the MSE of the reconstructed object. As can be seen, our joint reprojection approach quickly converges on alignment parameters, so that it requires fewer SIRT iterations to arrive at the same object MSE.

Figure 5 demonstrates the comparison of convergence plots of the prior sequential iterative reprojection approach (Algorithm 1), and our joint approach (Algorithm 2). The plots are obtained by calculating the relative mean-square-error between successive iterations. We separately calculated the errors in transverse (solid line) and axial (dashed line) directions. For the sequential approach, we used 40 iterations of the SIRT algorithm to reconstruct an estimate of the object before performing the alignment step, and we re-iterated this process 10 times, providing a total number of 400 reconstruction iterations. For the joint algorithm, we performed 400 SIRT iterations; however, this time we aligned the projection images with cross-correlation after each SIRT iteration. Our joint approach (Algorithm 2) provided superior convergence results for both the alignment estimation and the object reconstruction. We observed that the convergence rate of the axial alignment is more rapid than in transverse alignment. This is probably because of the fact that parallel-beam tomographic data are coupled in the transverse direction (where objects off of the axis of rotation move from projection to projection), while features remain at a constant position in the axial direction.

**Figure 6:**
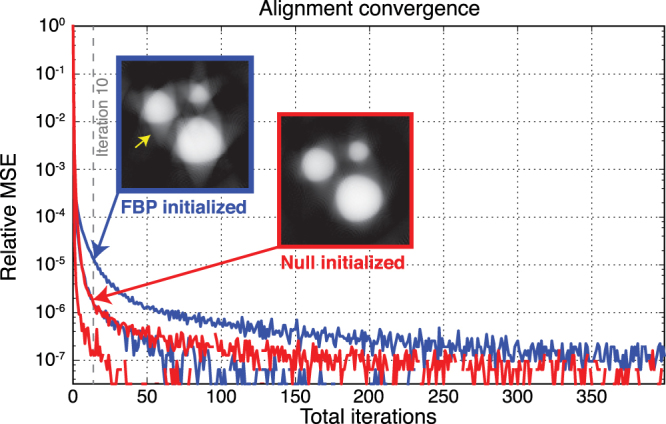
Convergence rates of joint alignment algorithm with different initializations. Blue and red plots corresponds to the relative mean squared error (MSE) in axial (rapidly convergent plot) and transverse (slowly convergent plot) alignment parameters for each successive iteration with filtered backprojection (FBP) and with null initialized algorithms, respectively. The reconstructed images after 10 iterations are shown for both FBP- and Null-initialized algorithms.

The initialization of iterative algorithms usually has an impact of the convergence rates and the final solution. In x-ray tomography, it is common practice to use filtered backprojection (FBP) results as the initial object estimate for iterative algorithms. Therefore, we examined the convergence rates of the joint alignment algorithm with different initializations as shown in Fig. 6. Blue and red plots correspond to the relative mean-square error (MSE) in the axial (rapidly convergent plot) and transverse (slowly convergent plot) alignment parameters for each successive iteration with FBP and with null initialized algorithms, respectively. Once again, axial alignment showed faster convergence than transverse alignment for both initializations. We observed that the convergence rate of the FBP-initialized algorithm is slower than the null-initialization. This is potentially because FBP provides strong streaking artifacts with misaligned projections and when too few rotation angles are used according to the Crowther criterion; this can make the algorithm be trapped in a local solution or need more iterations to recover from a false initial point. This is more evident by looking at the initial reconstructed images after 10 iterations, where the FBP-initialized algorithm produces artifacts on spherical objects, while the the Null-initialized algorithm produces smooth representations of spherical objects as expected.

**Figure 7:**
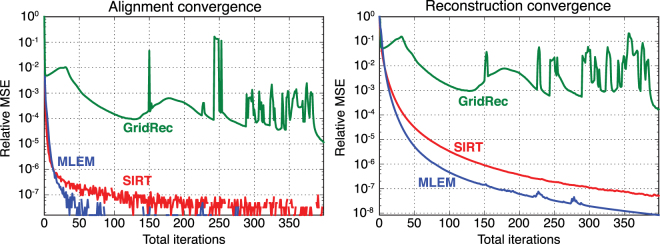
Comparison of three different reconstruction algorithms with respect to convergence rates for the alignment and object estimations with our joint algorithm as applied to our simulated dataset of Fig. 2. As can be seen, MLEM provides the most rapid convergence though with slight increases around iteration 225 and 275, SIRT provides a steady decrease in mean squared error (MSE), and GridRec (a filtered backprojection-type or FBP-type approach) provides poor performance for this dataset with insufficient rotation angle sampling according to the Crowther criterion^6^.

We also evaluated the effect of the choice of the reconstruction algorithm on the convergence rates in Fig. 7. We compared two common iterative methods – SIRT, and maximum-likelihood expectation-maximization (MLEM)^60^ – and a common non-iterative method, GridRec^61^. GridRec is a direct Fourier-based method that relies on discrete Fourier transforms of data in a manner similar to the filtered backprojection (FBP) algorithm. The main difference of GridRec with the FBP is that it samples a slice in the Fourier domain on a Cartesian grid before transforming it back to the spatial domain^62^. The utilization of fast Fourier transforms on a Cartesian grid outperforms other methods in terms of computational speed and is usually desired for quick reconstructions. We used a noise-free simulated object, and performed 400 joint algorithm iterations. MLEM provided the best results and the alignment procedure converged in less than 100 iterations, although subsequent iterations further improved the reconstructed object. The joint estimation of both alignment and reconstruction also cause some cross-talk between the convergence rates. For example, at the 220th and 275th iterations in MLEM we notice sudden increases in MSE due to changes in the alignment estimates, which we believe are due to numerical inaccuracies. The joint algorithm with GridRec failed to estimate both alignment and object, potentially because of the drift of the center of rotation at each iteration step, to the point where after 10 iterations the corrected translations were so large that the reconstructed object started to drift away from the field of view. It is well-known that Fourier-based methods such as standard filtered backprojection and GridRec do not work well when one has too few projection angles (thus violating the Crowther criterion, so that much of Fourier space does not contain projection data), so it is not surprising that both SIRT and MLEM outperform GridRec in our case of using 100 rotation angles with 100 × 100 pixel projection images.

**Figure 8:**
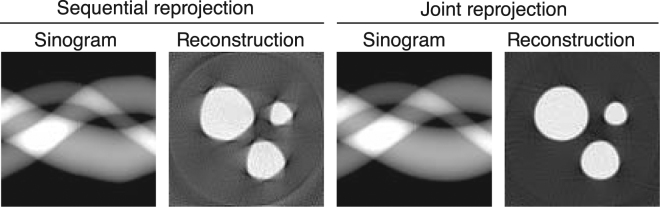
Reconstructed sinogram and corresponding tomographic reconstruction after the convergence of both the sequential (Algorithm 1) and joint (Algorithm 2) iterative reprojection approaches. As can be seen, our joint method produces a better reconstruction of the simulated dataset of Fig. 2 after 400 iterations.

We compared the reconstructions after the convergence of both the sequential (Algorithm 1) and joint (Algorithm 2) iterative reprojection approaches in Fig. 8. Both showed reasonably good quality sinogram reconstructions; however, the sequential algorithm introduced some reconstruction artifacts due to slight mismatch in estimating alignment parameters. These artifacts were almost nonexistent in the joint reprojection algorithm.

### Experimental validation

**Figure 9:**
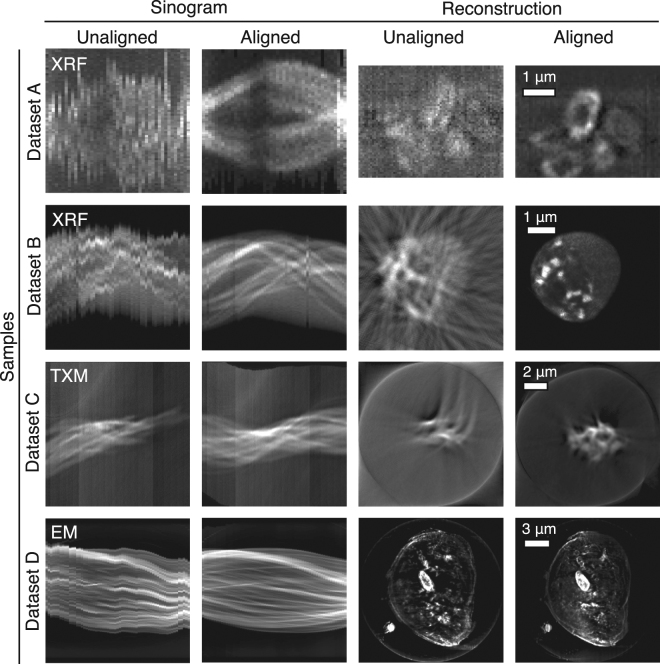
Sinograms, and reconstructed object slices, for the four different experimental datasets described in the Methods section (XRF = x-ray fluorescence, TXM = transmission x-ray microscopy, and EM = electron microscopy). The severity of the alignment errors in these tomographic datasets can be seen from the “wiggles” in the unaligned sinograms, and the poor quality of the reconstructed images. Our joint iterative reprojection reconstruction method provides the alignment information to remove these “wiggles” in the sinograms, and the reconstructed images are of much higher quality.

Having characterized the performance improvements of our joint iterative reconstruction approach (Algorithm 2) on simulated data, we then used it to obtain object slice reconstructions from the four experimental datasets: three from various x-ray microscopes, and one from an electron microscope. Sinograms and reconstructed object slices are shown in Fig. 9 both before and after the application of our joint iterative reprojection approach for compensating alignment errors. The severity of the alignment errors in each of the nanotomography datasets can be seen in by the “wiggles” in the unaligned sinograms as compared to the aligned sinograms, and the improvement in reconstructed image quality is evident.

**Figure 10:**
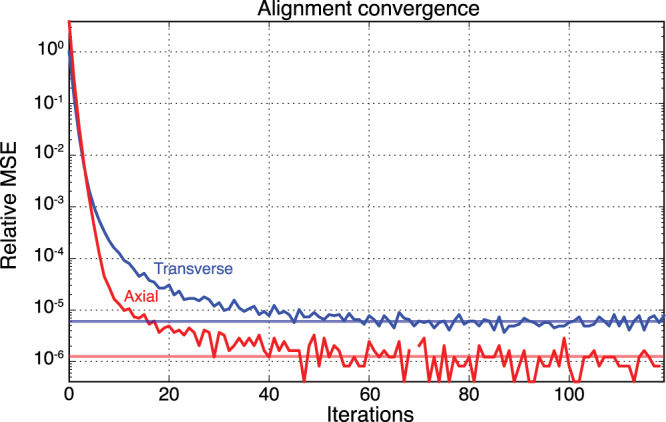
Convergence plots of the joint (Algorithm 2) iterative reprojection approach as applied to Dataset B, one of the experimental datasets. The blue and red curve show the relative mean squared error (MSE) for each successive iteration of the algorithm for transverse and axial alignments, respectively.

Figure 10 shows the convergence rates of transverse (blue) and axial (red) alignment parameters using the experimental Dataset B. The axial alignment convergence was faster than in the transverse direction; however, both converged after about 50 iterations. The convergence rates are similar to those we observed in simulation studies as shown in Fig. 5.

## Discussion

Tomographic reconstruction of the 3D object from the sinogram data requires precise knowledge of the object’s center of rotation with respect to the detector positioning. If this is not the case, systematic ring-like imaging artifacts appear in the reconstructed images, particularly at sharp boundaries, even when there is no geometrical distortion during data acquisition. The center of rotation can typically be determined by using various algorithms at a post-acquisition phase^63–66^. With the proposed joint algorithm we did not see any incorrect-centering artifacts in the reconstructions presented in this paper. This is probably because the back/re-projection operator enforces object updates at the center of the detector field-of-view, and automatically corrects the rotation center within its iterations. However, for cases, when the rotation center is significantly off, there is the possibility that the algorithm can fail to recover an object due to excessive blurring in intermediate object updates of the algorithm. In these situations, one of the above-mentioned algorithms can be used either at intermediate steps of the algorithm or after the alignment procedure to ensure that the rotation center used for back/re-projection is in the close proximity of the true rotation center.

In conclusion, we present here a rapid algorithm (Algorithm 2) for jointly aligning datasets and reconstructing the object. The algorithm avoids iterations in iterations, and can converge significantly faster than the previously used approach (Algorithm 1). We have characterized our joint iterative reprojection approach in simulations where the actual alignment errors were known, and we have shown that it works well when applied to experimental data. Given that many x-ray and electron nanotomography datasets are affected by nanometer-scale alignment errors caused by imperfections in rotation stages, this approach provides a path to obtain improved 3D reconstructions. Our initial demonstration presented here considered only translation errors in the individual projection images. We expect that image rotation errors can also be corrected in a similar manner, and that one can also correct for errors between the assumed and actual specimen rotation angles in the tomographic dataset. We plan on studying these extensions of our approach in subsequent work.

## Methods

### Problem definition

Here, we describe the mathematical basis of the alignment problem for 3D tomography. Let $f(x,y,z)$ be an unknown 3D object to be recovered. This can be expressed as a vector $f=[{f}_{1},{f}_{2},\ldots ,{f}_{N}{]}^{T}$, where $N={N}_{1}\times {N}_{2}\times {N}_{3}$ is the total number of voxels in $f$. Let the vector ${p}_{i}$ represents a 2D projection image with $M={M}_{1}\times {M}_{2}$ pixels captured at the ${i}^{th}$ tilt angle ${\theta }_{i}$. Ideally (without any translation error), the measurement process for each projection can be represented by,

1 $${p}_{i}={W}_{i}({\theta }_{i})f+{e}_{i},\quad i=\mathrm{1,}\ldots ,S,$$

where ${p}_{i}$ and ${W}_{i}({\theta }_{i})$ are, respectively, the recorded projection data and the projection matrices for the ${i}^{th}$ tilt angle, and $e$ is the additive measurement noise during the acquisition process.

Now we consider the translation model. The 3D object *f* can freely translate in the $x$, $y$, and $z$ directions, which leads to a 2D translation error over the projection images. Therefore, in our model, we apply the translation error to the 2D projection images instead of the 3D object (translation errors in the $z$ direction have no effect in standard parallel-beam tomography). The measurement geometry and translations are given in Fig. 1. The ${i}^{th}$ projection image can be described as ${p}_{i}={T}_{i}({h}_{i},{v}_{i})W({\theta }_{i})f$, where ${T}_{i}$ represents an $M\times M$ geometrical transformation operator, and ${h}_{i},{v}_{i}$ are unknown transverse and axial translation errors for ${i}^{th}$ projection image, respectively. If we stack the $S$ projection images, Eqn. (1) can be rewritten in a compact form as,

2 $$p=T(h,v)W(\theta )f+e,$$

where $p=[{p}_{1},{p}_{2},\ldots ,{p}_{S}{]}^{T}$ is the vector of size $MS\times 1$, holding the tomographic measurement dataset, $T(h,v)={\rm{diag}}[{T}_{1}({h}_{1},{v}_{1}),{T}_{2}({h}_{2},{v}_{2}),\ldots ,{T}_{S}({h}_{S},{v}_{S})]$ is the geometrical translation matrix of size $MS\times MS$, and $W(\theta )=[{W}_{1}({\theta }_{1}),{W}_{2}({\theta }_{2}),\ldots ,{W}_{S}({\theta }_{S}{)]}^{T}$ is the matrix of size $MS\times N$ for the measurement process. Here $h=[{h}_{1},{h}_{2},\ldots ,{h}_{S}{]}^{T}$ and $v=[{v}_{1},{v}_{2},\ldots ,{v}_{S}{]}^{T}$ are unknown transverse and axial translation errors for all $S$ projection images respectively, and $\theta =[{\theta }_{1},{\theta }_{2},\ldots ,{\theta }_{S}{]}^{T}$ denotes $S$ tilt angles during the acquisition process.

**Algorithm 1:**
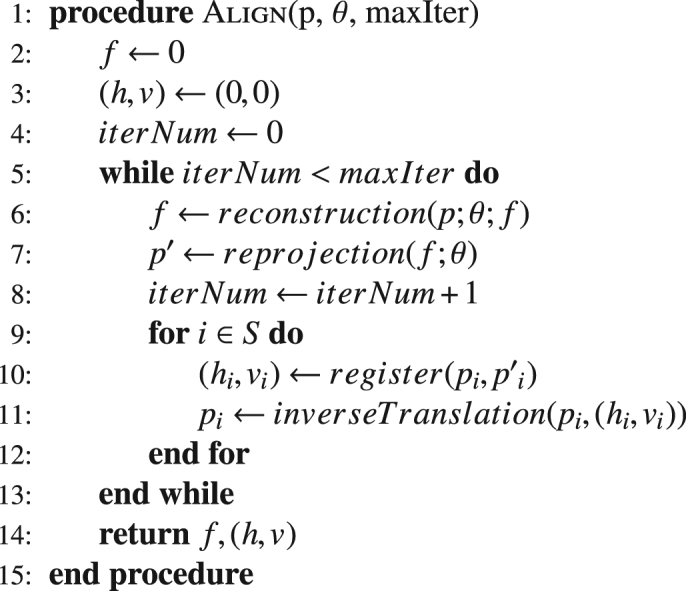
Proposed 3D joint alignment and reconstruction.

The goal in the iterative reprojection tomography reconstruction is to recover both the translation error $(h,v)=({h}_{1},{v}_{1};{h}_{2},{v}_{2};\ldots ;{h}_{S},{v}_{S})$ and the 3D object $f$ simultaneously in a joint fashion. The most common way to achieve this is to minimize the distance between the real captured projection data and reprojection data calculated from the current reconstruction. Algorithm 11 summarizes the details of the proposed method.

### Implementation details

We implemented our new approach (Algorithm 11) in Python while drawing upon openly available libraries. We used the open-source and documented package TomoPy^67^ for the reconstruction and reprojection operators, and the open-source Scikit-Image library^68^ for calculating phase-correlation based sub-pixel image registration^69^ and image warping operators. The analytical phantoms and corresponding simulated measurements were generated using Xdesign^70^, which is another open-source Python package for simulating x-ray data.

### Experimental datasets

Dataset A is of X-ray fluorescence data collected at the Bionanoprobe (BNP)^71^ at the Advanced Photon Source at Argonne National Laboratory. This instrument uses a double crystal Si 111 monochromator to produce 10 keV incident x-rays, and a Fresnel zone plate to produce a focus spot with a theoretical Rayleigh resolution of 85 nm. A silicon drift detector was placed at 90° with regards to the incident X-ray beam direction to collect fluorescence signals from which element-specific images were obtained using the analysis program MAPS^72^. A tomographic dataset was obtained using projection images of 79 × 56 pixels at 60 nm pixel size and a dwell time of 100 msec per pixel, over a rotation angle range of 0° to 135° at 3° increments (0° refers to the sample plane being perpendicular to the incident X-ray beam). This dataset represents the X-ray fluorescence from a cluster of dried bacterial cells with a metallic surface tag imaged at room temperature; the maximum signal was about 50 detected photons per second.

Dataset B is of Gadolinium x-ray fluorescence data collected at the Hard X-ray Nanoprobe (HXN) Beamline^73,74^ at the National Synchrotron Light Source II (NSLS-II) of Brookhaven National Laboratory. As with the Bionanoprobe, a 10 keV beam was monochromatized, a Fresnel zone plate was used to produce a focus spot with a theoretical Rayleigh resolution of 49 nm, and a three-element fluorescence detector was placed at 90° to the incident beam to collect fluorescence signals from which element-specific images were obtained using PyXRF (https://github.com/NSLS-II/PyXRF), which is an equivalent elemental mapping software such as MAPS. A tomographic dataset was obtained using projection images of $200\times 120$ pixels at 25 nm pixel size and a dwell time of 30 msec per pixel (with a maximum Gd signal of about 450 detected photons per pixel), over a rotation angle range of −90° to +90° at 3° increments. The sample used was a ceramic composite with mixed ionic and electronic conductivity^75^, consisting of a Ce_0.8_Gd0.2O_2_ (CGO) oxygen ionic conductive phase and a CoFe_2_O_4_ (CFO) electronic conductive phase. Nanostructure at the grain boundary is expected to have significant impact on the conductivity of this material^75^.

Dataset C is of 8 keV x-ray transmission data collected at the Transmission X-ray Microscope (TXM) of sector 32-ID at the Advanced Photon Source^76^. This microscope uses a specialized diffractive condenser optic to illuminate the sample, and a Fresnel zone plate objective with an experimentally measured resolution of 60 nm to produce a magnified image on a scintillator/lens/CCD image detector system. A tomographic dataset was obtained using projection images of $2160\times 2560$ pixels each at 15 nm pixel size and a per-projection acquisition time of 1 second, over a rotation angle range of −90° to +90° at 0.25° increments. The dataset was then downsampled $4\times$ to $540\times 640$ pixels, from which a $200\times 200$ pixel subregion was extracted at each projection angle for tomographic reconstruction. The 32-ID TZXM is equipped with an air bearing rotation stage with $\sim 1.25$ $\mu$ rad wobble and an eccentricity of $\pm 100$ nm over 360°, so this dataset had fewer initial alignment errors than the x-ray fluorescence datasets A and B though thermal drifts can still affect specimen translation over the 20 minute data acquisition time. The sample used was a cathode particle agglomerate (Li_1.2_Co_0.1_Ni_0.15_Mn_0.55_O_2_) of a Lithium-ion battery with volume of about 6 $\mu m{}^{3}$.

Dataset D is of 200 kV high-angle annular dark field (HAADF) data acquired using a Hitachi HD2300 scanning transmission electron microscope at the Northwestern University Atomic and Nanoscale Characterization Experimental Center (NU*ANCE*). A tomographic dataset was obtained using projection images of 256 × 256 pixels at 66 nm pixel size and a per-pixel dwell time of 10 $\mu$sec, over a rotation angle range of −60° to +60° at 1° increments. A nano-diffraction beam setting was used in order to maintain sufficient depth of focus. The specimen imaged was a human buccal cell collected utilizing a Cytobrush and deposited on a TEM grid. The sample was chemically fixed with glutaraldehyde (2.5%) and formaldehyde (2%) in PBS for 20 minutes. After fixation, the sample was gently washed and then plunge-frozen in liquid ethane (Vitrobot Mark III, FEI) and turbo freeze dried (K775X, Emitech). The dry sample was then plunge-frozen again, transferred to a cryo single tilt holder (Gatan) and kept at liquid nitrogen temperatures during imaging.

### Data availability

The algorithms used here are made publicly available in the TomoPy package at https://tomopy.readthedocs.io. The simulated and experimental datasets used are available in TomoBank at https://tomobank.readthedocs.io.
